# Community nurse-paramedics’ sphere of practice in primary care; an ethnographic study

**DOI:** 10.1186/s12913-021-06691-y

**Published:** 2021-07-18

**Authors:** Tuija Rasku, Marja Kaunonen, Elizabeth Thyer, Eija Paavilainen, Katja Joronen

**Affiliations:** 1 Faculty of Social Sciences, Health Sciences, Tampere University, 33014 Tampere, Finland; 2 General Administration, Tampere University, Pirkanmaa Hospital District, 33014 Tampere, Finland; 3 Dean’s Unit School of Health Sciences, Western Sydney University, Locked Bag 1797, Penrith, NSW 2750 Australia; 4 General Administration, The Hospital District of South Ostrobothnia, 60220 Seinäjoki, Finland; 5 Department of Nursing Science, University of Turku, Joukahaisenkatu 3-5, 20520 Turku, Finland

**Keywords:** Health services, Primary health care, Community paramedicine, Sphere of practice

## Abstract

**Background:**

Primary care, the principal function of the health care system, requires effort from all local primary health care teams. Community Paramedicine (CP) has managed to reduce the use of Emergency Medical Services (EMS) for non-emergency calls, but for the paramedic to move from traditional emergency calls to non-emergency care will mean new demands. There is a paucity of research exploring nurse-paramedics’ experiences and perceptions of their novel roles as community paramedics in Finland. This study aims to explore the community nurse-paramedics’ (CNP) experiences in their new sphere of practice.

**Methods:**

A descriptive ethnographic study was conducted, to collect data through participant observation (317 h total) and semi-structured interviews (*N* = 22) in three hospital districts (HD) where the CNPs have worked for at least 1 year. Both data sets were combined, organised, and analysed using inductive content analysis.

**Results:**

Five main categories were developed by applying inductive content analysis: the new way of thinking, the broad group of patients, the way to provide care, the diversity of multidisciplinary collaboration, and tailored support from the organisation. The CNP was identified as needing an appropriate attitude towards care and a broader way of thinking compared to the traditional practice of taking care of the patient and the family members. The diversity of multidisciplinary collaboration teams can be a sensitive but worthwhile topic for offering new possibilities. Tailored support from the organisation includes tools for future CP models.

**Conclusions:**

Our results indicate the CNPs’ deep involvement in patients’ and families’ care needs and challenges with their skills and competencies. Their professional attitudes and eagerness to develop and maintain multidisciplinary collaboration can offer preventive and long-term caring solutions from which citizens, allied health, safety, and social care providers benefit locally and globally.

## Background

Primary Care is a pivotal and principal function in the health care system [1]. It is the first contact care; its providers are expected to be comprehensive, and the care needs must be coordinated. Primary Care is person-focused (not disease-oriented) care provided as close to the user as possible, where diseases may cause medical, psychological, or social problems. National primary care strategies need efforts from local primary health care teams. Health services are tailored to meet the populations needs, aiming to establish a continuum of care and contribute to its comprehensiveness [2]. There is an urgent need to create new health care models in the primary care setting to decrease pressure on home-care providers’ (e.g., home care nurses and professional caregivers) [3, 4].

Internationally, CP is community-engaged, patient-centred preventive care that includes multidisciplinary collaboration and has many programmes as an integrated part of the primary care setting [5]. To work as a community paramedic means that the paramedic steps out from the traditional emergency role instead of providing patient assessment and non-emergency care with allied prehospital health care providers. In the future, many health care providers’ roles might change, and the necessary competencies could be broader, but the care should remain focused on quality and safety [4, 6].

As primary health care endeavours to follow these changes, it is faced with increased demands, which lead to a greater need for allied health professionals to carry out assessments and treatment traditionally delivered by physicians [7]. In some prehospital jurisdictions, paramedics work seamlessly with the allied health care professions, providing the patients with well-organised and high-quality care [4–6, 8–11].

Globally, CP programmes have filled the gaps in local health care delivery and assessed the possibilities for patients to remain at home. The community paramedics have assessed patients, taken advanced blood tests, analysed ECGs, arranged referrals to X-ray or follow-up visits, assisted with medication management, or arranged social support [7, 12, 13]**.** They have been supported through protocols or have been directed online (telephone or video) by relevant physicians, e.g., geriatricians, general internists, family practitioners, paediatricians, or emergency physicians [14]. The reports and evaluations of CP programmes have underpinned the need for organisational support, long-term planning of the multidisciplinary collaboration and challenges to the economy, or the reimbursement of the provided health care service [14, 15].

The Finnish government regulates customer-oriented and supportive health care services [16]. According to their proposal, one-person EMS units could provide non-emergency patient assessment and minor treatments beyond the emergency services provided by ambulance units. These one-person Finnish EMS units are comparable to CP units around the world. In Finland, EMS staff are mostly nurse-paramedics. Nurse-paramedics undertake 4 years of full-time studies at a university of applied sciences (including 4450 h of theoretical studies and 2025 h of practical training) following the European Union requirements. In the first year of “nursing studies”, Finnish nurse-paramedics learn about care for people with chronic conditions concentrating on emergency care in the following years. The graduated nurse-paramedic will be registered as a nurse, and he/she will work under the direct supervision of the EMS physician of the HD [16]. The CNPs’ role has developed in response to the shortage of health care providers in primary care and the awareness of their independent skills in assessing patients in challenging circumstances.

Generally, the traditional views regarding the paramedics’ role and the competencies they need have changed. In previous studies, paramedics were associated with many different roles, such as clinicians, team members, health and social advocates, educators, reflective practitioners, and professionals. These roles were linked with patient safety, compassion, adaptability, and communication [12, 17]. There is a paucity of research exploring nurse-paramedics’ experiences and perceptions of their novel roles in Finland. By conducting an ethnographic study in the context of CNPs, we aim to identify and explore the actions, experiences and perceptions of the Finnish CNPs’ sphere of practice. By analysing their responses, we add to the growing body of research into CP, contribute to this health care model’s possibilities, demands, and sustainability, and provide policymakers, health care providers, and educators with essential information for future use.

## Methods

### Methodology

The study aimed to explore the Finnish nurse-paramedics’ experiences and perceptions of their work as CNPs. We used an ethnographical research framework because ethnography aims to deepen the understanding of social phenomena in a social setting [18–20]*,* and the ethnographic approach is especially recommended in cultural changes from one stage to another [21]. We wanted to know how the Finnish CNPs experienced changing from a nurse-paramedic to a CNP. Ethnographic research orientation includes fieldwork, the position of the researcher, and the purpose of the study. The researcher is the essential research “device” when the researcher is personally among the participants [20].

Our study design for data collection included participant observation and semi-structured interviews. When taking part in people’s lives, listening to their words, and interpreting their actions, we could collect a “thick description” [18], meaning that the researcher reflects social events and actions, and the description and analysis are rooted in reality. Participant observation is the recommended method for gaining an in-depth understanding of a phenomenon [18, 19]. It revealed nuances from the CNPs’ daily work, which would not have come to light in interviews alone [22]. The researcher needs to understand the participants’ perceptions (the “emic” view), which helps uncover the reasons for their actions. Health researchers have used the outsider’s perspective (the “etic” view) when trying to identify and describe patients’ problems instead of listening to the patients’ (“illness experts”) own ideas of their condition, feelings, and perceptions. In an ethnographic study, both emic and etic views, are needed as thy summarise. The participants’ insights and words into the language of science [20]. The consolidated criteria for reporting the qualitative research (COREQ) -checklist was used to mitigate the inherent threat of bias and report the essential aspects of the research [23]. An ethics approval was obtained from Tampere University Hospital Ethics Committee (Approval R19008H).

### Study setting

Finland is divided into 21 hospital districts (HD) to provide specialised medical care. Every HD has a central hospital and municipal health care centre associated with a university hospital. The research was undertaken in three HDs in different parts of Finland. The size of the HDs was similar (population ranged 130,000 – 190,000; area 7000–22,000 km^2^). These HDs were selected purposely because the CP model ran in them for longer than 1 year. Each HD has one or two CP units providing non-emergency health care services with other health care providers and safety workers such as the police and the safety alarm responders. The nurse-paramedics working as community nurse-paramedics have extensive experience in emergency or prehospital nursing. They have undertaken additional training on risk assessment (risk of chronic disease and falling), assessing the patient’s mobility and social needs, advanced diagnostics, medication management (e.g., antibiotics), simple procedures (e.g., suturing), and palliative care.

### Study participants

We used purposive sampling, including 13 CNPs, four nurse-paramedics, four team leaders, and one care coordinator (*N* = 22). Purposive sampling is commonly used in qualitative research [20]. The participants received the study invitation and provided informed written consent via email. No participants refused or dropped out. The key participants were from different HDs to give a multifaceted understanding of this health care model and the CNPs’ roles, foci and perceptions. All key participants had worked as CNPs since the model was first implemented and hold specialised and expert knowledge about the related history and interaction processes [21].

### Data collection

The data collection began in May 2019 and continued until September 2019. The researcher (TR) gathered the data through observations, informal discussions and semi-structured interviews in each HD. Participant observation consisted of riding along with the CNPs, observing their practice, and talking to staff members and co-workers (home care nurses, care coordinators and doctors). The observation (317 h) occurred across all HDs in the same summer and by one researcher (TR) to mitigate the inherent threat of bias. The researcher (TR) had no relationship with any participants before the study. Thirteen CNPs, four team leaders, four nurse-paramedics, and one care coordinator participated in the semi-structured interviews.

This multi-method approach captured the diversity of the CP models within a natural setting and allowed the issues to be studied in-depth. The length of the observational period and the number of interviews were not pre-determined; they continued until the thematic saturation was met.

The participants, patients, and family members were informed about the purpose of the study and the researcher’s role. They were reminded that each participant could withdraw at any time, and they would stay anonymous in the data. During visitations, the CNP asked the patient (or the family member or home care nurse if the patient could not speak or understand the question) if the researcher could stay. The patient or the CNP could deny the researcher’s presence at any time. The researcher did not take part in the interventions. The observational period included the CNPs’ patient assessments by phone or face-to-face visits, training sessions with other health care providers, meetings with allied health care workers, and cooperation with the police and safety alarm providers.

The researcher (TR) spent between two to five 10˗12-h shifts with each CNP. There was 1 day between the work shifts while the researcher transcribed the field notes and the interviews. Afterward, in the second round, the researcher (TR) returned to the interviewees to confirm the findings. The observational notes and transcripts were read and discussed with the research team members to ensure the consistency of the interpretation. The researcher (TR) kept a field diary, where contemporaneous descriptions were extended and detailed after each workday. Informal discussions with the CNPs were an essential component of the observation part of the data collection process. During the observation, the CNPs’ practice was noted immediately after patient calls, along with the CNPs’ comments.

**Fig. 1:**
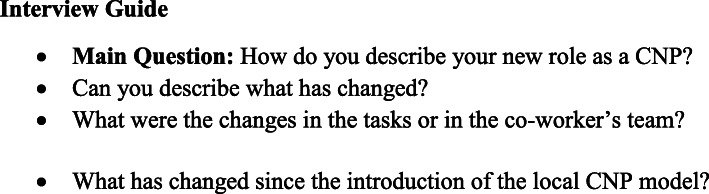
Interview Guide of Community nurse-paramedics

The observational data were completed with semi-structured interviews. The interviews were conducted following an interview guide (Fig. 1) to ensure that the researcher collected similar data from all participants and gained all their perspectives.

The questions were piloted with two nurse-paramedics and one team leader (led by TR) to assess clarity and potential bias. No changes were needed. The data from the pilot interviews were not included in the analysis. The interviews were conducted with 13 CNPs, and the data were enriched by interviewing four nurse-paramedics, four EMS team leaders, and one care coordinator (*N* = 22).

The interviews were a mix of formal and informal, which is a common practice in ethnographic research [20]. Four of the CNPs and two of the team leaders (involved with the CP model) were on holiday, and because of the distances, they were interviewed by phone. Formal interviews were audio-recorded and later transcribed verbatim, whereas informal interviews sometimes evolved from conversations and were recorded using contemporaneous notes and further descriptions immediately after the interview. The data were organised, the fieldnotes were sorted, and the interviews were transcribed. The interviewees were provided with the “transcript” during the next work shift, and corrections were made as needed [20]. The transcripts were anonymised and stored in a password-protected database.

### Data analysis

**Fig. 2:**
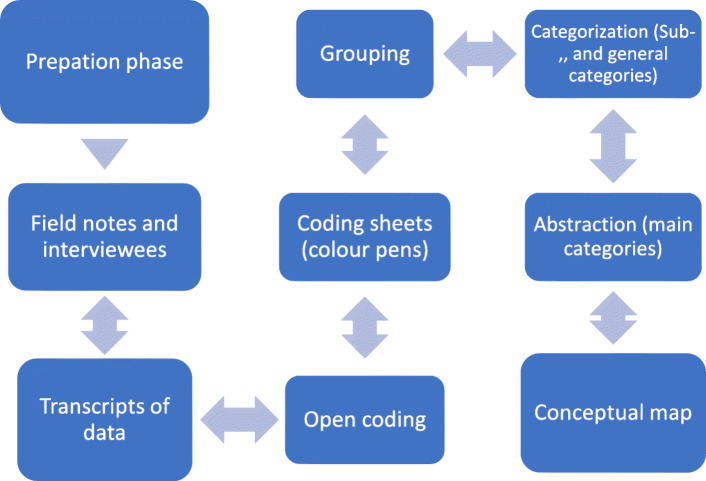
The phases in the inductive approach of the content analysis process Modified from Elo&Kyngäs (2008)

The data collected using both methods were combined and analysed using inductive content analysis. The inductive content analysis process includes open coding, coding sheets, grouping, categorisation, and abstraction [24]. (Fig. 2).

When words and phrases are classified into the same categories, they share the same meaning [25]. With open coding, notes and headings were added to the text while reading it. The researcher read the written material three times (TR) to avoid the risk of focusing too much on some parts of the data. Reflecting on the meaning of the data allows the codes to emerge from the data. New headings were written in the margins to describe various aspects of the content. Thus, the researcher did not lose sight of the meaning and depth of findings [20].

Two other researchers (KJ and MK) joined the analysis with no prior knowledge of the data. The researchers frequently discussed the coding process and clarified any disagreements in the codes. The original expressions yielded open codes (*N* = 213). The codes were coloured to better identify closely linked material better. After the open coding was completed, the categories provided a means of describing the phenomenon [25]. Each subcategory was labelled using content-characteristic words and then grouped into generic categories, which were further grouped into main categories [24]. Any inconsistencies were resolved by joint review and discussion. Original citations increase the trustworthiness of the research and determine what kinds of original data categories are formulated [26]. Table 1 presents an example of the inductive content analysis process.

**Table 1:** Example data extracts demonstrating the hierarchical coding process

Original text	Open codes	Subcategory	Generic category	Main category
*“..it is important that you* ***sit down with the patient and the family members*** *and try* ***to find the solution****, which I think means that there is some* ***long-term plan*** *for the person* ***to survive at*** *home ...”*	sitting down with the patient & the family finding the solution making the long-term plan	time to sit patient & family solutions long-term planning survey at home	the luxury of time together with the patient & family the aim of the assessment the aim of the care	the way to provide care

## Results

In this study, 22 health care professionals participated, 10 of which were female and 12 male. Of the participants, there were 13 CNPs, four nurse-paramedics, four EMS team leaders, and one care coordinator. The participants had all worked longer than 5 years as health care providers and since the introduction of the local CP model. The field period was a total of 317 h, which provided a” ‘thick description” of the research area [18]. During the field period, the researcher observed 12 CNPs’ practices during 94 patient assessments.

**Fig. 3:**
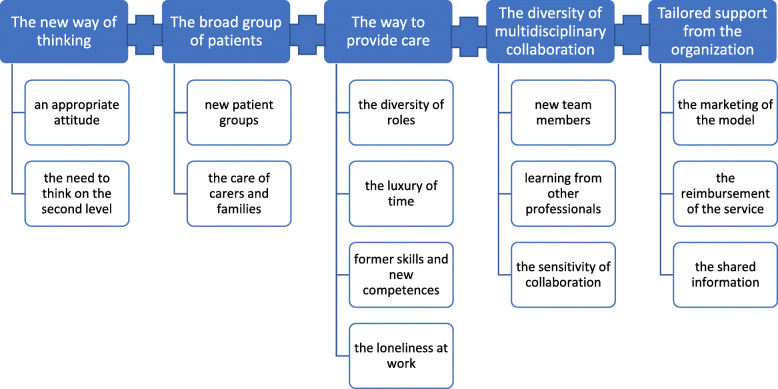
The Community nurse-paramedics’ novel sphere of practice

The data from the interviews and observational field notes produced 213 open codes, categorised into 56 subcategories and then to 14 generic categories. The initially identified categories were reduced, and five categories were developed from the data: (1) The new way of thinking, (2) The broad group of patients, (3) The way to provide care, (4) The diversity of multidisciplinary collaboration, and (5) Tailored support from the organisation (Fig. 3). Each category included coded quotations to illustrate the findings.

### The new way of thinking

The interviewees described the CNP’s new way of thinking, including *an appropriate attitude* and *the need to think on the second level*.

The CNPs articulated that working as a CNP means a readiness to change the mind from lights and sirens and episodic care into the search for long-term solutions for the patient. The interviews highlighted that the CNP has an appropriate attitude when they voluntarily engage in the new role. The most significant risk is when the CNP behaves wrongly. For various reasons, it could destroy the relationship between the patient and the family and the relationships in the multidisciplinary collaboration team.

*Not all of us can work as CNPs. This job is for you when you do not get kicks anymore from the lights and sirens. You must be ready to concentrate on the patient and the family for a longer time than before. In the beginning, it could be very hard.* (Excerpt 1, CNP)

The comments related to *the need to think on the second level* indicated that CNPs do not think differently, but they need to think more broadly than before, trying to find a continuum of care with other multidisciplinary team members.

*The CNP got a phone call from the home care assistant. Her client, a 92-year-old lady, lives alone and now has low blood pressure. The CNP assessed the patient’s care needs by phone and found that the mentioned blood pressure was normal for her from her previous patient records. The lady could stay home but she wanted to go to the shop because the fridge was empty. The CNP advised the lady not to go to the shop and called a food delivery service to provide food for the lady. The service will deliver food for the lady immediately, and the home care assistant promised to make an extra visit to the lady in the evening.* (Excerpt 2, Observational notes)

### The broad group of patients

The interviewed CNPs characterised the broad group of patients as *new patient groups* and *the care of carers and families.*

The CNPs emphasised that most CP patients are old and live either alone or with older or frail spouses. They provide more palliative care to patients of different ages than before and have discharged patients or patients with their families as their new patient groups. The CNPs felt that the need for social support is most often behind many physical considerations.

*All of our patients are not clients of home care. A discharged patient might have a wound that could be open and secreting, but the patient does not have any pain medication or is too scared to clean the wound or even go to the shower. The spouse can be very scared and not sleep at all. If we are not visiting there, we (EMS) might have two patients in the morning. We try to explain what “spray the wound” or “take a painkiller when needed” means during our visit.* (Excerpt 3, CNP)

*We visited a male patient in the elderly care home. He was very drowsy during dinner. The CNP assessed the patient checked the urine sample and read from the patient’s history that he has had a couple of urinary tract infections. The patient has a urine infection again, and after consulting the doctor-on-call, the CNP arranged the medication from the local pharmacist. The CNP called the elderly care home in the evening, and the patient felt much better.* (Excerpt 4, Observational notes)

CNPs can deliver advanced diagnostics, and they have received additional education on palliative care. With these patients, the CNPs help the at-home nursing mostly at night and on weekends when home care nurses are unavailable. The patients have a direct number to the CP unit.

*On Christmas Eve, I called the palliative care physician directly. We decided that the patient could stay at home over Christmas and did not need to go to the hospital to change the medication. I organised it with the local pharmacist, and the family was pleased.* (Excerpt 5, CNP)

The CNPs received positive feedback when the patient could avoid the uncomfortable travel to the ED and back, for instance, to control the PCA (patient-controlled analgesia) pump. The CNPs felt that the relationships between the patients and the families were equally rewarding and demanding.

*I see more smiles than before. The patients and the families are relieved to remain at home. If I had eaten all that chocolate and cakes that these grateful patients or family members offered, I would be 30 kg heavier.* (Excerpt 6, CNP)

The CNPs noted that their job does not end after the ambulance has taken the patient to the ED. They also take care of the carer (spouse or family member) left at home. The spouse might need help surviving at home while the patient is in hospital. Sometimes the “healthier” spouse needed transportation to the hospital, and the CNP organised a place for them to stay overnight.

*The spouse was taken to the hospital, and I saw that the wife was very pale. I sat down with her and asked how she was coping. She started to cry and told me that she was fatigued. They did not have anyone to come, so I managed to get a temporary place for her to rest. Before this CP model, we would have taken the husband to the hospital and ask that the wife cope at home.* (Excerpt 7, CNP)

### The way to provide care

The interviewees described the CNP’s expansive provision of care with *the diversity of roles*, *the luxury of time, former skills and new competencies,* and *loneliness at work.*

The CNPs described having many roles while delivering care. They were advocators, navigators, innovators, developers, therapists, and repairers while working as health care professionals. Their patient assessment started from the front yard rather than upon meeting the patient. They sometimes felt that they worked between the patient and society.

*The main thing is not to fix the patient; we like to connect them to the right place and the right people. One idea could be day-care visiting clubs for older adults to see other people because so many are just lonely.* (Excerpt 8, CNP)

The CNPs articulated that the most positive addition and enabler to providing care is *the luxury of time*. They can sit down, talk with the patient, and there is no need to rush. Time enables them to obtain a more holistic picture of the patient and the family and offers possibilities to find longer-term solutions.

*It is peaceful to sit down on the sofa and discuss the whole family's solutions and challenges. It gives me a deeper meaning in our work if we try to find a solution for tomorrow.* (Excerpt 9, CNP)

The interviewed CNPs felt that they experienced a significant demand to rehearse their *former nursing skills*. At the same time, they needed new competencies like providing telephone triage and assessing the patient’s fall risks, nutrition level, or signs and symptoms of sepsis.

*I have not used some nursing skills since school, like urine catheterisation. Now we need to practice them. Another challenge is that we have excellent blood test machines, but we need to understand more than just the numbers they give us.* (Excerpt 10, CNP)

The interviewees emphasised that a large part of their work is telephone triage. Home care nurses consult them, and they consult different specialists or general physicians, and rarely EMS doctors. A substantial number of phone calls include arrangements for the doctor’s patients’ care and appointments.

*Today we visited only one patient, but the CNP got phone calls all day. Most of the calls came from home care, and the CNP assessed the patient’s care needs, gave nurses advice, or contact numbers, or organised appointments at the Health Care Centre. Some phone calls sounded more like coaching the family member to cope with the patient.* (Excerpt 11, Observational notes)

The interviewees were concerned about maintaining their former emergency skills and still working in the CP unit.

*The job rotation between the CP unit and the ambulance unit is excellent. Now I can keep in touch with the emergencies and with these more like psycho-social-physiological CP visits.* (Excerpt 12, CNP)

The CNP’s work is very independent, but it comes with a degree of *loneliness*, which could be very hard in the beginning. The CNP works alone and makes the decisions alone. Sometimes he/she must advise the patient and his/her family of bad test results facing their sadness and disappointment at their home.

*The CNP must be ready to make the right decision and make it there, in front of the family and alone. This job has its loneliness. When you work as a nurse-paramedic, you can talk with your partner during the assessment and immediately afterward, but now you are alone. The consultant doctor is in the hospital, not in front of a little child when you need to tell the family about the decision, which may destroy their trip to the zoo.* (Excerpt 13, CNP)

### The diversity of multidisciplinary collaboration

The interviewees described the diversity of their multidisciplinary collaboration with *new team members, learning from other professionals,* and *the sensitivity of collaboration.*

The CNPs worked more intensively with other health care providers and the police than before. Their work teams also included home care nurses and assistants, safety alarm responders, specialised nurses such as psychiatric nurses, and different specialised doctors, including EMS doctors-on-call.

*Nowadays, we work as the partner for the police patrol. They do not take the driver to the ED for the alcohol level or drug blood test. They call us, and we meet at the police station. The guardian is there with us, and the patrol is free to go back for their actual job. If these people were taken to the ED, as in the old days, these patients could be restless and noisy, so it is a safety matter as well, you know.* (Excerpt 14, CNP)

Through multidisciplinary collaboration, the CNPs were able *to learn from other health care professionals*. For example, if the CP call included information that the patient might have mental health difficulties, the CNP picked up the psychiatric nurse from the ED, and then both professionals assessed the patient at home.

*I must admit that when I followed how the psychiatric nurse was talking with the patient and how she ends up with the solution, it was something that you cannot learn at school or from the books. The psychiatric nurses want to learn the ABCDE approach when we are there, and I have learned a lot about motivational interviewing from them.* (Excerpt 15, CNP)

*Today I and a home care nurse decided that we will go together to the patient, who has a new kind of catheter. The nurse taught me how to work with it. Now it is nicer to visit the patient as I saw how the home care professionals are taking care of it.* (Excerpt 16, CNP)

As part of the cooperation, the CNP may telephone the patient and speak with them to relays information about the situation, the patient’s condition, or medical history to the ambulance unit.

*When the CNP calls us about the patient’s pre-information, it is much safer to go to the patient. We can use the driving time more efficiently when we have information about the patient’s health history.* (Excerpt 17, Nurse-Paramedic)

The interviewees spoke about *the sensitivity of the collaboration*, especially concerning rumours and misunderstandings harming the multidisciplinary cooperation. Some respondents were concerned about how other health care providers would react or if they felt threatened by the CP model and how the CNPs’ provide care.

*So quickly, I think that home care nurses felt that we are taking their place initially. It was not valid. I think the assumptions were easily wrong. It is our job to convince others that we are working together. When possible, I just exchange even some informal words with the home care nurses or social care providers – breaking the ice, you know*. (Excerpt 18, CNP)

### Tailored support from the organisation

The participants indicated three support-related factors for the successful implementation and future of CP models. The identified supporting factors were *the marketing of the model*, *the reimbursement of the service*, and *the shared information* (told and documented).

The CNPs underlined that navigating the fragmented health care system requires clarity and more management than before. The interviewees described the need for powerful *marketing of the model* to the community, co-workers, and citizens.

*This new cooperation is compassionate. In the beginning, somebody should adequately inform people about who we are and what we do – the rumours can harm a lot. Our co-workers could be scared and make assumptions easily. The next thing is that we need a new reimbursement system. It is too much like the old EMS way, where the money comes based on the kilometres driven, not based on the care delivered.* (Excerpt 19, CNP)

The interviewed CNPs pointed out the urgent need *for the shared information* about patient records. Some CNPs could check the patient’s hospital records to obtain information about the background about their condition. Some CNPs could use only the patient records from primary care, and others had no access to patient records. The lack of a standard patient record system forces the CNP to plan and decide the patient’s care without the necessary information. The CNPs highlighted the importance of the essential and intensive consultation with the physician, which enables receipt of the required information and a guarantee of the patient’s continuum of care.

## Discussion

This study explored the Finnish CNPs’ experiences and perceptions of their CP care and identified the following critical elements: (1) the CNPs’ attitudes need to be appropriate, and their way of thinking needs to be on a new level; (2) the carer of the family member is added to the group of patients; (3) the CNPs’ possibilities to provide care are broad; (4) the diversity of multidisciplinary collaboration can require sensitivity; and (5) the CNPs highlighted essential tailored support from the organisation for the future of the CP model. Our findings help fill the gap in the current literature about how CP providers perceive their work.

In this study, we found that the core of the CNP’s new way of thinking was the appropriate attitude towards non-emergency care and the ability to think broader than before: “on the second level”. Furthermore, the willingness to work as a CNP, possessing excellent interpersonal skills, and having developed extensive experience were highlighted. The result is consistent with a previous study that underlined that not all paramedics want to or should become Community Paramedics [27, 28]. The willingness to participate in a CP programme should be considered during the selection of new nurse-paramedics. The interviewed CNPs highlighted excellent interpersonal skills and wide-ranging experience in their work as critical competencies required for practicing CP.

Our results indicated that the broad group of CP patients included mostly elderly and multimorbid patients living at their own homes or elderly care homes. Internationally, CP programmes for elderly citizens have been successful, and the participants of these programmes have a low risk of returning to the ED or being readmitted to the hospital [29–31]. Additionally, the Community Paramedics have taken part in additional training for palliative care, and the new role has been rewarding [32]. Our study is the first known study where police clients were also included as the CNPs’ patients. When the police patrol can avoid visiting the ED with a drunk driver and instead take the driver directly to the police station, it benefits all participants and may further increase safety for others in the ED.

Patient assessment at home is a unique situation, and other family members (the spouse and/or children) play an essential role in enabling the patient’s stay at home; sometimes, the spouse or family member needs more support to cope with the responsibility [33–35]. Patients are discharged from the hospital very early, and the family caregiver, sometimes a very old spouse, is given a responsibility that could create an emotional, physical, or financial burden [36]. The CNPs were aware of some spouses’ fatigue or fear of being left at home with a frail spouse in our results. A recent study [37] discussed caring for carers and how informal care may result in a “spill over” effect when the pressure is excessively placed on the shoulders of the spouse. Our findings illuminate that the CNPs’ patient assessment is now more holistic because they have time to assess the patient and consider the strengths of the other family members or environmental risks (“...now we can check the fridge, kitchen tables, and toilet…”) before trying to find a solution. The assessment as a part of the provided care has more dimensions than a primary survey conducted as a nurse-paramedic. The ABCDE approach in the CNPs’ primary survey should be expanded to include an F for Future, Family, or Friends as sometimes the actual story or history (e.g., how the patient is coping at home or over the past few days) is heard from someone other than the patient.

This study illuminated how CNPs provision of care is particularly demanding, including different roles, enablers, and challenges. Our findings are consistent with several previous studies that revealed that paramedics have various roles, such as clinicians, health and social advocates, team members, educators, professionals, or reflective practitioners [8, 12, 17]. Based on our findings, the CNPs mostly assumed clinical or health advocacy roles. While delivering care as a clinician, the CNPs use their knowledge, skills, and clinical judgement within the given scope of practice. Advocacy, speaking on behalf of the patient [38], increases the demands on the CNPs as they are challenged to find long-term solutions for how to enable the patient to remain at home as long as possible or how to reach the right place to meet the patient’s care needs. According to our study, the participating CNPs did not have just a dual role between emergency and non-emergency services [13], but at least a quadruple role.

The CNPs highlighted the luxury of time as one of the cornerstones to providing functional care. The unhurriedness has been noticed as a significant enabler for client-centred home care services [35, 39]. Our results are consistent with this and signify the CNP’s possibility of settling down and spending additional time finding more solutions for the patient’s care needs. More time with the patient is also an essential motivator for the CNP to work. Peaceful time with the patient enables home screening and ensures care that includes the medical, social, and psychological assessment of the patient and the family, finally arriving at a better solution to cope at home. In a previous study, the paramedics indicated that the chance to spend more time assessing and understanding patients’ unmet long-term needs created a disparity between their traditional values and the requirements of ambulance units to identify life threats and transport all patients without delay [40]. Unhurriedness is a new component in the CNPs’ patient assessment, and it can take time for a former nurse-paramedic to learn to use it correctly to provide comprehensive care.

The CNPs are familiar with patient assessment and with providing minor treatments or administering medication. In this study, the CNPs pointed out that they can rehearse their formerly acquired nursing skills with the community nurses, for example, in wound therapy and urine catheterisation. This finding is consistent with several previous studies investigating multidisciplinary practice in action and highlighting the importance of health professionals’ recognition of the needs for informal training with each other [41, 42]. To work in primary care requires specific capabilities and additional education for paramedics [43]. The professional knowledge of paramedics and nurses working in primary care has been compared [44] and the crucial role of education in the sustainable implementation of the CP model has been identified [45]. The Finnish CNPs highlighted a dual advantage in the need for multidisciplinary collaboration. The CNPs could rehearse their nursing skills with community nurses and, at the same time, have the chance to discuss the CNPs’ role and the aim of the CP model.

Based on our findings, the CNPs spent a lot of time on the phone consulting the doctor and home care providers. Telephone triage for patient assessment was one of the new and necessary competencies mentioned by the interviewed CNPs. After similar results, some institutes have added this “phone work/telemedicine” into the Community Paramedics’ tailored training [46]. The effectiveness of phone assessment has been highlighted in previous research. One-third of people calling the triage nurse were satisfied with the care instructions or guidance from social and health care services [47].

Furthermore, telecare offers new possibilities for patients, especially in rural areas, and it enables other health care providers to advise citizens safely by phone, for instance, during COVID-19 and other outbreaks of contagious disease [48]. Furthermore, the CNP used phone assessment to obtain pre-information before the ambulance unit was sent to the patient. Working by phone contributed to the provided health care for the patient and the caring team members.

The independent nature of the work and the diversity of possible solutions for the patient’s needs can create feelings of powerlessness. In our study, the CNPs described that sometimes the independence of the work made them feel lonely. These findings have implications at the organisational level because emotions like loneliness can cause exhaustion, distress, and lower job satisfaction [49], although this is more likely if the employee feels that the work is also without meaning [50]. The CNPs pointed out that previously, there was always another person with whom they could share thoughts and decisions in the ambulance unit. The physical distances between the patients can be very long, and patient visits frequently keep the CNP on the road throughout their shift without seeing any other co-workers during the day. One rewarding, personally relevant, and emotionally salient solution has been for team leaders to meet the team members [50] frequently and to actively share feedback, keeping them engaged in their integrated teams [51]. However, paramedics have stated that they rarely received feedback from patient interactions [40]. In our findings, on the contrary, the CNPs articulated that they received direct and positive feedback almost daily, which is one of the necessary drivers against pervasive feelings of loneliness. Other remedies for loneliness included the luxury of time with the patient and the possibility for long-term solutions. As proposed in this study, the CNPs’ possibilities to provide care are broader. However, based on these results, the independent nature of the work creates challenges that must be considered at the managerial and organisational levels.

All participants highlighted the diversity of multidisciplinary collaboration. Collaboration has been defined as one of the keys to the success of the CP programme [5, 52]. The CNPs spoke about the multidisciplinary team and its social and cultural context with the possibility to learn from each team member. The CP teams have included midwives [41], pharmacists [53], and social workers [54]. Our results show that the teams are growing with other EMS providers, staff from the ED, home care nurses and assistants, psychiatric nurses, the police, personal safety alarm responders, and specialised physicians. Nurse-paramedics have a long history of collaboration with EMS doctors-on-call, and in the CP model, the essential partner can be a general practitioner [55]. The interviewed CNPs underlined that the formal and informal meetings with co-workers are essential for receiving and giving the correct information and building up appropriate expectations about the collaboration. The meeting could be an informal meetings according to a previous study [56]; these findings are consistent with our results. The CNPs were worried about the correctness of information about their sphere of practice. They felt that collaboration could be compassionate when different professions work on the same “playground”. The expectations of the ED staff can quickly create misinformed perceptions. The CNP models contribute to the health care system. However, health care management and organisation must provide clear and correct foundational knowledge about each of the roles and responsibilities.

Our study identified essential parts of the tailored support from the organisation for CNPs and the CP model. The interviewees specified challenges with planning, management, and reimbursement in the CP model. These results are consistent with previous studies [9, 14, 57, 58], in which the challenges of implementing the CP programmes with other health care models have been underlined. Rumours can harm the future of the model if the model’s marketing starts too late. CP funding needs to change to allow payment for care that does not involve the transport of patients to the ED [14, 15]. The need for an accurate exchange of patient information between CNPs and other primary, specialty, and emergency care providers, or the CNPs’ possibility of documenting the care directly in the official patient records enables the sharing of information. The need for the electronic transfer of health information is consistent with the results of previous studies [14, 59]. The opportunity to provide a patient assessment with broader information could improve the CNPs’ options for making long-term and correct decisions. Moreover, the availability of electronic prehospital information linked to hospital and primary care information (for example diagnosis) enhances continuous care for individual patients and possibilities for the system-level analyses of the outcomes-based EMS research.

The change from work as a paramedic in the ambulance unit to a one-person CP unit creates challenges and advantages. The dimensions of the new sphere of practice influence the CNPs’ job satisfaction, work motivation, and wellbeing. More research is needed on the role, foci and perceptions of the allied health and safety professionals and how they see the future of CP.

### Limitations

This study is not without limitations. First, the study was conducted in three Finnish HDs, which, although diverse, are not representative of all CNP models. Therefore, as in ethnographic research, the results represent an interpretation of the observations, experiences, and stories. It is possible that everything was not captured during the observation periods or in the interviews. However, the principal researcher (TR) moved between HDs for the observation periods to address this. Secondly, purposive sampling may lead to bias [20]. However, this sampling ensured that the interviewees have the relevant background.

Thirdly, the data analysis and coding were performed by a single researcher who was also an experienced emergency nurse-paramedic. The researcher’s background allowed for assuming the emic perspective to understand the insider’s perceptions. The emic perspective is essential in an ethnographic study for a researcher to explain events from the participant’s perspective [20]. The long CNP visits allowed the interviewees and the researcher to elicit their views. The authors discussed the quotes, avoiding presenting raw data without analysis. The quotes of the participants in this study are not raw data but purposefully selected by the researchers.

## Conclusions

This descriptive ethnographic research of Finnish CNPs provides insight into how the CNPs perceive and experience their novel sphere of practice. The change from emergency and episodic care to non-emergency and holistic care need to be voluntary for the CNP. Professional attitudes, interpersonal skills, and the ability to build and maintain teamwork are essential for nurse-paramedics undertaking this new practice line. Working independently and very closely with the patient and their family gives the CNPs positive feedback and deeper involvement in their lives. Nonetheless, it can be lonesome. The CP collaboration team is broad, and it could challenge the CNPs’ social skills, but it also offers the possibility to learn new skills and practice the previously learned competencies. However, CNPs also face challenges (for example, the shared information and reimbursement of the service) that compete with their desire to serve patients and, if left poorly addressed, may threaten the sustainability and future of the CP model. This study includes essential knowledge for health and social care providers, policymakers, and educators when planning and developing the CP model. Further studies are needed to explore the expectations and experiences of the CNPs’ co-workers, the CP patients, and families,’ which could enhance the implementation of the CP model in communities locally and globally.

## Data Availability

The data that support the findings of this study are not publicly available according to the ethical approval from the Regional Ethics Committee of Tampere University Hospital as it contains information that could compromise research participants privacy and confidentiality. It is deemed that even when no names are attached to the data, the participants may be able to be identified through the interview and observation transcripts which are the data source for this study.

## Abbreviations

ED: Emergency Department
EMS: Emergency Medical Services
CNP: Community nurse-paramedic
CP: Community paramedicine
HD: Hospital District
